# The mitochondrial genome of an important edible insect species, the African palm weevil (*Rhynchophorus phoenicis*)

**DOI:** 10.1080/23802359.2024.2342929

**Published:** 2024-05-07

**Authors:** Benjamin J. Roberts, Doubi Bi Tra Serges, Konan Konan Jean Louis, CM (Tilly) Collins

**Affiliations:** a Georgina Mace Centre for the Living Planet, Imperial College London, London, UK; b Marc Delorme Research Centre, Centre National de Recherche Agronomique (CNRA), Abidjan, Côte d'Ivoire; c Centre for Environmental Policy, Imperial College London, London, UK

**Keywords:** *Rhynchophorus phoenicis*, African palm weevil, mitochondrial genome, edible insect, food security

## Abstract

The African palm weevil (*Rhynchophorus phoenicis*) is a species of high economic importance in sub-Saharan Africa, both as a culturally traditional edible insect and as an agricultural pest. Here we provide a *de novo* assembly and annotation for the mitochondrial genome of this species from whole-genome sequence data. The mitogenome was AT-rich and 17,161bp in length, containing 13 protein-coding, 22 transfer RNA, and two ribosomal RNA genes. Phylogenetic reconstruction showed the African palm weevil to cluster within the genus *Rhynchophorus* and the weevil tribe Rhynchophorini. This mitogenome will be important for future genetic research into this emerging edible insect species.

## Introduction

The African palm weevil (*Rhynchophorus phoenicis*, Fabricus 1801; Coleoptera: Curculionidae) is a large beetle found across sub-Saharan Africa. The species is both a severe pest of palm plantations and an important edible insect for human consumption, being popularly eaten across Africa (van Huis 2013). When raised on palm waste, weevil larvae can form a highly nutritious foodstuff (Anankware et al. 2021), circularizing palm cropping cycles in the process. Therefore, it is a key candidate for promoting sustainable food security in Africa and has high socioeconomic importance.

Despite this importance, genetic resources for this species are lacking. Thus far, mitochondrial and nuclear genomes are unprofiled and population genetic structure across Africa is unknown. Here we assemble and annotate the first mitochondrial genome for this economically important species. It is hoped that this resource will play a key role in future genetic research, which is expected to become increasingly prevalent as captive breeding of the species becomes more widespread across sub-Saharan Africa.

## Materials and methods

**Figure 1.**
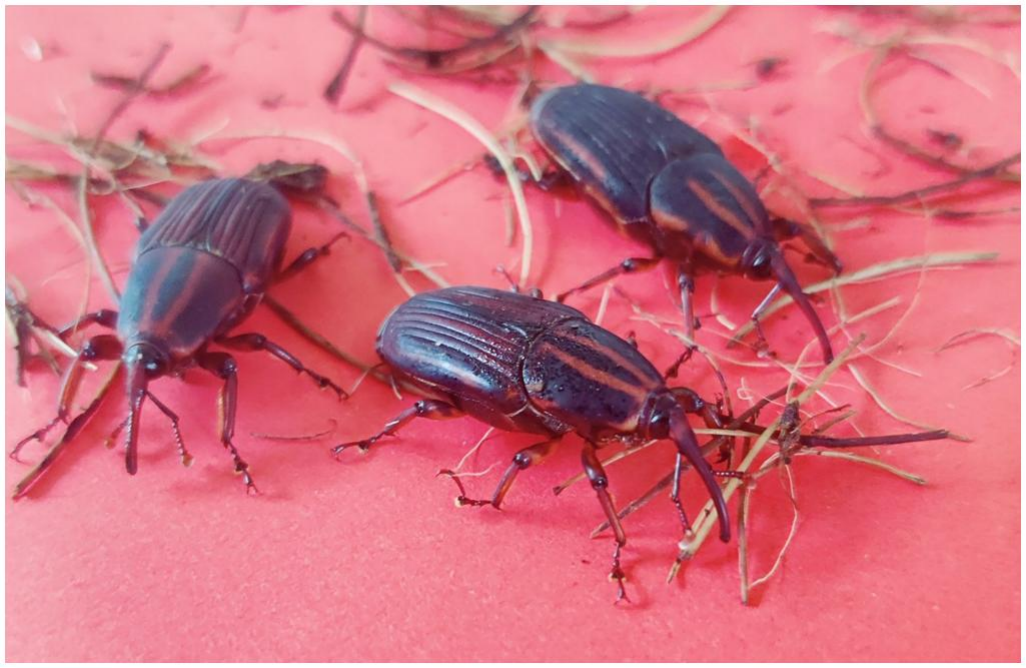
Three adult individuals of *Rhynchophorus phoenicis* (photo credit BJR; OnePlus 8, f/1.8, 1/1000). The species generally reaches body lengths, including the rostrum, of 3.5–4.5 cm (Tanyi Tambe et al. 2013).

Nine adult individuals were collected in September 2022 from the Marc Delorme Coconut Research Station (5.282058, −3.8420376) in Port-Bouët, Côte d'Ivoire. Specimens were deposited at Imperial College London, Silwood Park campus under accession number VS9815 for downstream analyses (Ben Roberts, b.roberts21@imperial.ac.uk). A species reference image for the species is shown in Figure 1.

Total DNA from a single individual was extracted using a Qiagen DNeasy blood & tissue kit (Qiagen Ltd., Manchester, UK), which was whole-genome sequenced on an Illumina NovaSeq platform (GENEWIZ, Leipzig, Germany). Paired-end 150 base pair (bp) reads, using 1.8 gigabases of raw whole-genome sequence data, were assembled, annotated, and visualized using MitoZ according to the methods of Meng et al. (2019).

For phylogenetic analysis, the new assembly was combined with nine insect mitochondrial genomes obtained from the NCBI database (Sayers et al. 2022); these comprised eight other species of Coleoptera and the honey bee, *Apis mellifera* (Hymenoptera), as an outgroup (Table 1). Species were selected from the available mitochondrial assemblies to span the weevil genus *Rhynchophorus* and the sub-family Dryophthorinae, both containing the African palm weevil, as well as non-Dryophthorinae members of the weevil family Curculionidae. Two widely used non-weevil beetles, *Megasoma elephas* and *Oryctes rhinoceros*, were also selected to provide taxonomic range. The sequences were aligned in MUSCLE (v3.8.31; Edgar 2004) and a maximum likelihood phylogenetic tree was produced in RAxML (v8.2.9; Stamatakis 2014), using the GTR + G + I evolutionary model and 1000 bootstrap replicates, and visualized in iTOL (v6; Letunic and Bork 2021). The optimal model of sequence evolution was chosen by Akaike information criterion in MegaX (v10.2.6; Kumar et al. 2018).

**Table 1.** Publicly available mitochondrial genomes used in the phylogenetic analyses.

Species	GenBank accession number	Citation
*Rhynchophorus phoenicis*	OR466183	This study
*Aclees cribratus*	NC_051548	Wang et al. (2020)
*Alcidodes juglans*	NC_041669	Xu et al. (2019)
*Apis mellifera*	NC_051932	Unpublished
*Megasoma elephas*	OK484310	Ayivi et al. (2021)
*Oryctes rhinoceros*	NC_059756	Unpublished
*Rhynchophorus ferrugineus*	KT428893	Zhang et al. (2017)
*Sipalinus gigas*	NC_053351	Unpublished
*Sitophilus oryzae*	NC_030765	Ojo et al. (2016)
*Sitophilus zeamais*	NC_030764	Ojo et al. (2016)

## Results

**Figure 2.**
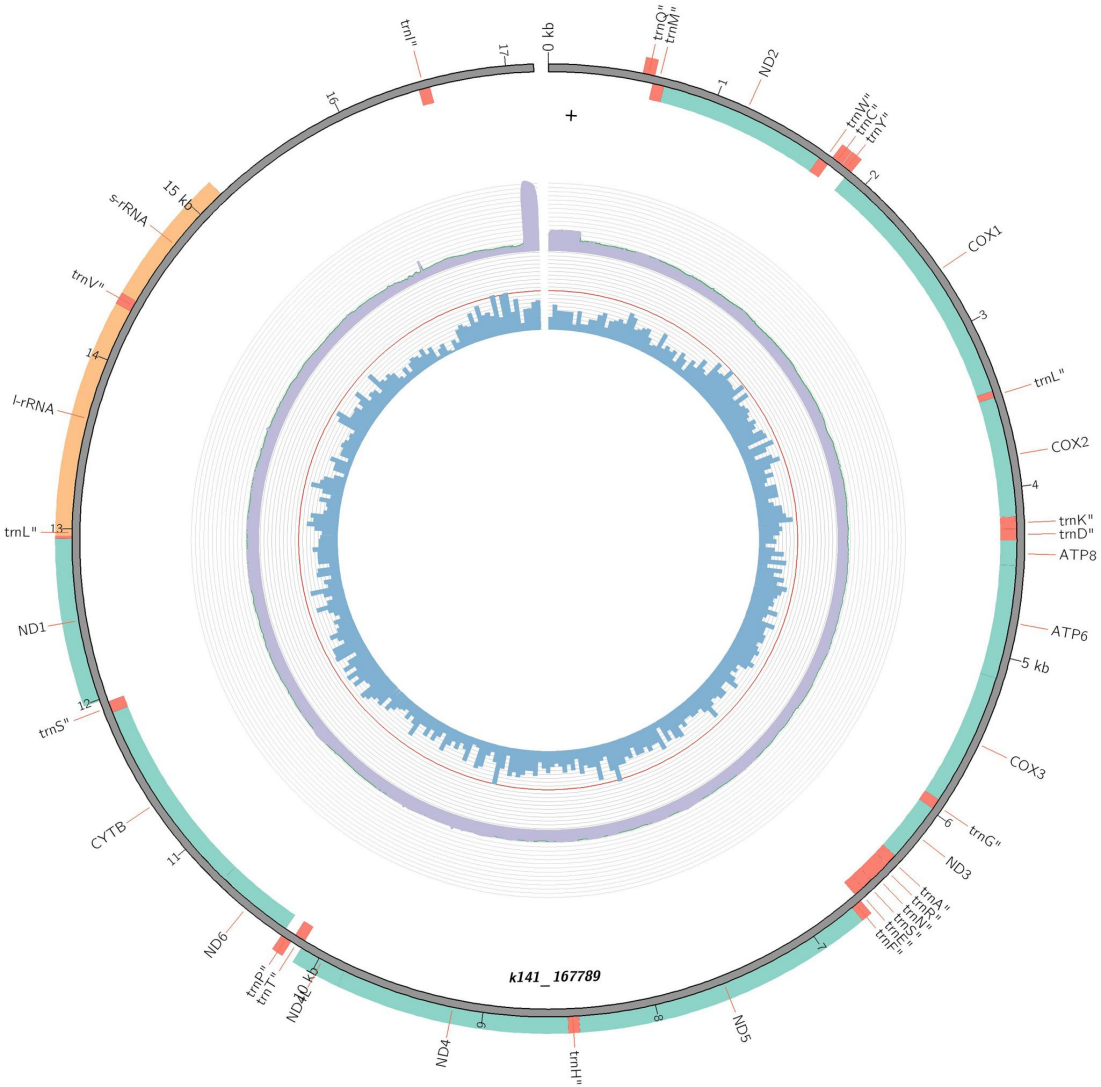
The mitochondrial genome of the African palm weevil (*Rhynchophorus phoenicis*). The 13 protein-coding genes, 22 tRNA, and two rRNA genes are shown in green, red, and orange, respectively. This plot was produced in MitoZ (Meng et al. 2019). GC content, in 50 bp windows, is shown by the blue bars, with the red ring that of 50% GC content, and genome-wide depth coverage is shown by the purple ring.

The new mitochondrial genome (GenBank Accession: OR466183) was 17,161 base pairs in length. The genome contained 13 protein-coding, 22 transfer RNA, and two ribosomal RNA genes (Figure 2), in the same order and orientation as the ancestral insect (Cameron 2014). The composition was AT biased, comprising 37% A, 16% C, 10% G, and 37% T. Depth coverage for the assembly is shown in Supplementary Figure 1.

**Figure 3.**
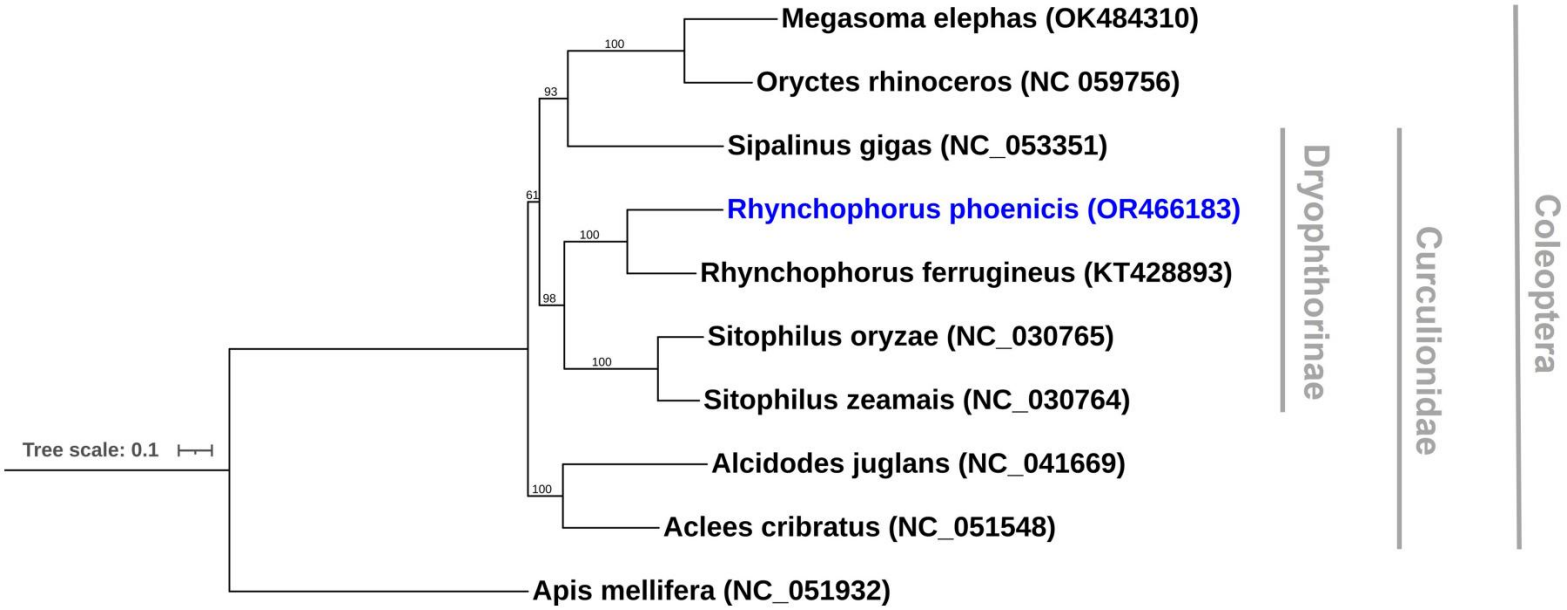
Maximum likelihood phylogenetic tree of nine coleopteran mitogenomes, including the new assembly produced here (in blue), and a single hymenopteran outgroup. Bootstrap support values, as a percentage of 1000 replicates, are given on nodes. GenBank accession numbers for publicly obtained sequences are shown in brackets and the papers they derive from are given in Table 1. Branch lengths are proportional to the number of substitutions per site (see scale bar).

Phylogenetic analysis showed the genus *Rhynchophorus* to form a clade, with the new assembly clustering with the only other available mitogenome from this group, the red palm weevil, *Rhynchophorus ferrugineus. Rhynchophorus* formed a sister group to the weevil genus *Sitophilus*, containing the maize and rice weevils. Both Curculionidae, the weevil family, and Dryophthorinae, a weevil sub-family containing the African palm weevil – often upgraded to family rank (Alonso-Zarazaga and Lyal 1999) – were resolved to be paraphyletic; two scarab beetles, *Oryctes rhinoceros* and *Megasoma elephas*, were found nested within them (Figure 3).

## Discussion and conclusion

The mitochondrial genome assembly provided here compares closely to other insect and beetle species. The genome size of 17,161 base pairs falls within the reported range for insects (Cameron 2014) and is comparable to other weevil species (Ojo et al. 2016; Zhang et al. 2017; Ma et al. 2020). Closely related weevil species also show similar AT biases in their mitochondrial genomes to that found here (Ojo et al. 2016; Zhang et al. 2017; Ma et al. 2020).

As expected, the assembly clustered with *Rhynchophorus ferrugineus* and the weevil genus *Sitophilus*; these two genera are members of the weevil tribe Rhynchophorini. Interestingly, the relationships within wider Coleoptera were less well resolved, likely due to the low taxonomic coverage and limited loci used in the phylogenetic analysis. The two scarab beetles, *O. rhinoceros* and *M. elephas*, clustered within the weevil family Curculionidae, and formed a clade with *Sipalinus gigas*, a member of the weevil sub-family Dryophthorinae. This disagrees with previous multi-locus phylogenetic reconstructions of beetles, which have both the families Curculionidae and Scarabaeidae as monophyletic (Shin et al. 2018; Zhang et al. 2018; McKenna et al. 2019). This indicates that additional genetic markers and/or greater taxonomic sampling are required to fully resolve these relationships.

Future genetic research into this important species should focus on resolving patterns of genetic diversity and divergence across the African continent, particularly in genes underlying economically important traits. The mitogenome presented here can play a key role in this, both in the design of species-specific amplification primers in future population genetic studies and as a high-quality reference for the West African population.

## Supplementary Material

Supplemental Material

Supplemental Material

## Data Availability

The genome sequence data that support the findings of this study are openly available in GenBank of NCBI at https://www.ncbi.nlm.nih.gov/ under accession no. OR466183. Associated BioProject, SRA, and BioSample numbers are PRJNA1037096, SRR26723342, and SAMN38162519, respectively.
