# Supplementary figures and images for: The mitochondrial genome of an important edible insect species, the African palm weevil (Rhynchophorus phoenicis)
Source: Mitochondrial DNA B Resour. 2024 May 7;9(5):601–4. doi: 10.1080/23802359.2024.2342929 (PMC11080666; doi:10.1080/23802359.2024.2342929)

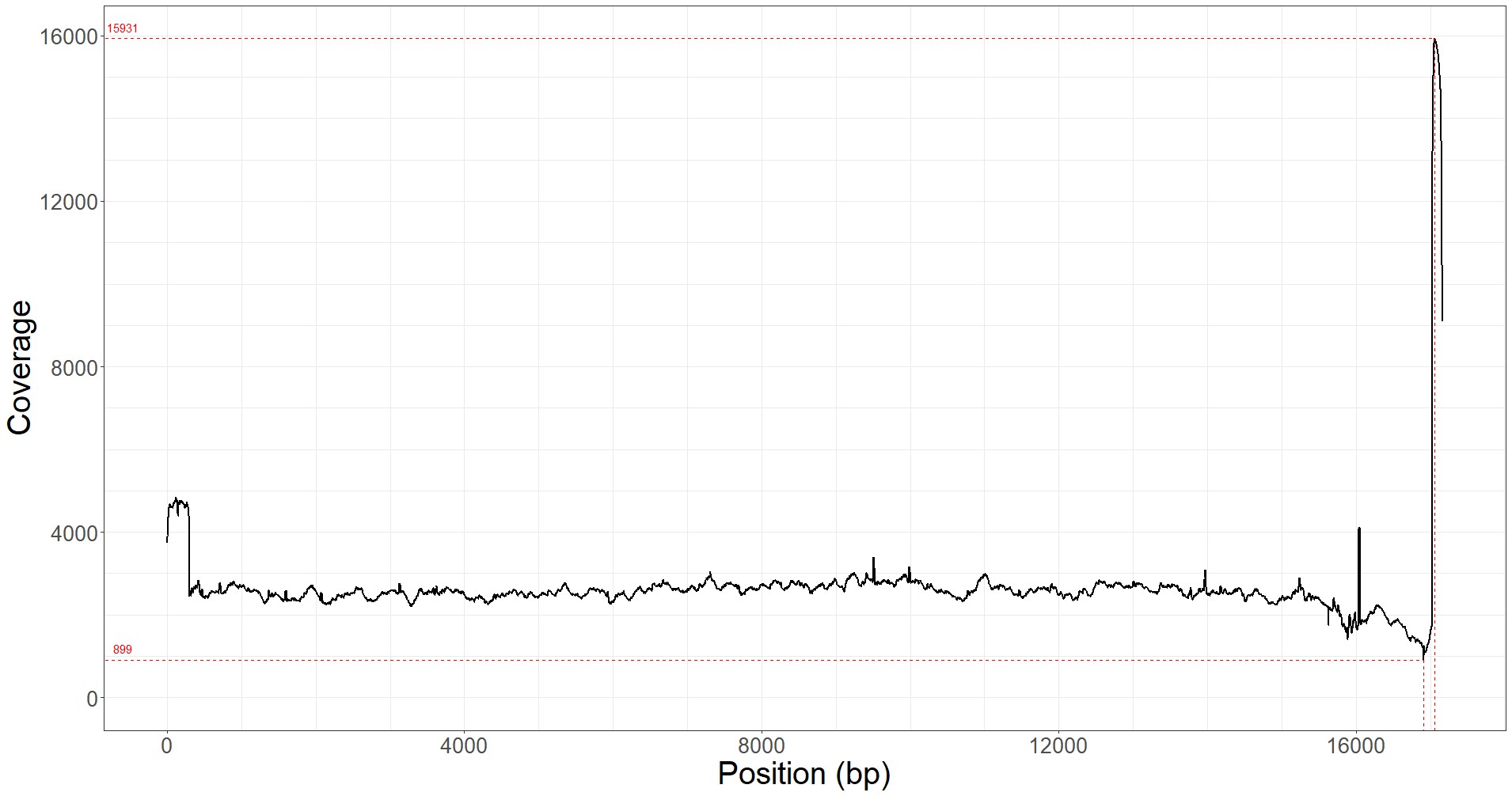

Supplement: Supplemental Material [file TMDN_A_2342929_SM9066.jpg]
